# Prebiotic selection for motifs in a model of template-free elongation of polymers within compartments

**DOI:** 10.1371/journal.pone.0180208

**Published:** 2017-07-19

**Authors:** Grant Kinsler, Sam Sinai, Nicholas Keone Lee, Martin A. Nowak

**Affiliations:** 1 Dept. Applied Mathematics, School of Engineering and Applied Sciences, Harvard University, Cambridge, MA, United States of America; 2 Dept. Biology, Stanford University, Stanford, CA, United States of America; 3 Program for Evolutionary Dynamics, Harvard University, Cambridge, MA, United States of America; 4 Dept. Organismic and Evolutionary Biology, Harvard University, Cambridge, MA, United States of America; 5 Wellcome Trust Sanger Institute, Wellcome Genome Campus, Hinxton, CB10 1SA, United Kingdom; 6 Dept. Mathematics, Harvard University, Cambridge, MA, United States of America; Santa Fe Institute, SPAIN

## Abstract

The transition from prelife where self-replication does not occur, to life which exhibits self-replication and evolution, has been a subject of interest for many decades. Membranes, forming compartments, seem to be a critical component of this transition as they provide several concurrent benefits. They maintain localized interactions, generate electro-chemical gradients, and help in selecting cooperative functions as they arise. These functions pave the way for the emergence and maintenance of simple metabolic cycles and polymers. In the context of origin of life, evolution of information-carrying molecules and RNA based enzymes within compartments has been subject to intensive theoretical and experimental research. Hence, many experimental efforts aim to produce compartments that contain elongating polynucleotides (also referred to as protocells), which store information and perform catalysis. Despite impressive experimental progress, we are still relatively ignorant about the dynamics by which elongating polynucleotides can produce more sophisticated behaviors. Here we perform computer simulations to couple information production through template-free elongation of polymers with dividing compartments. We find that polymers with a simple ability—biasing the concentration of monomers within their own compartment—can acquire a selective advantage in prelife. We further investigate whether such a mechanism allows for cooperative dynamics to dominate over purely competitive ones. We show that under this system of biased monomer addition, even without template-directed self-replication, genetic motifs can emerge, compete, cooperate, and ultimately survive within the population.

## Introduction

Understanding how biological information emerged and was maintained in the origin of life remains a key open question [1, 2]. In modern living systems, a key portion of inherited information is stored in a digital form within RNA and DNA molecules. For decades, RNA has been the primary candidate proposed to store and transmit information in early life [3–5]. This was postulated because RNA can act as a template for inheritance as well as a catalyst for a variety of critical reactions [6–9]. Motivated by this idea, known as the “RNA world hypothesis,” there has been ample experimental effort to find RNA molecules with polymerase or replicase activity [6–10]. Replicases (enzymes capable of replicating themselves or other similar molecules) have been of particular interest because they would commence Darwinian evolution immediately and, in principle, result in progressive increase in complexity [11]. Experimental studies have found impressive ribozymes capable of catalyzing particular reactions in template-directed replication [8, 10, 12, 13]. In particular relevance to our work, ribozymes with the ability to bias the presence (or production) of specific building-blocks have been investigated [14–19]. Nonetheless, generic prebiotically-plausible polymerases capable of efficiently catalyzing self-replication have yet to be found [20]. While replicases may be necessary, they are not sufficient for life to progress. The path from replicases to modern cells is not without obstacles. Eigen [21] has noted that replicases would quickly lose to their competitive “cousins” if they could not replicate their own template with high accuracy. This is known as the “error-catastrophe” problem. Furthermore, any cooperative enzymes in a well-mixed environment may be exploited by parasitic molecules that benefit from the cooperative function but do not reciprocate. Such dynamics could also lead to quick elimination of cooperators [22–28].

Thus, there is also interest in non-enzymatic or template-free models of growth and evolution in early life dynamics [13, 29–33]. Independent theoretical approaches from thermodynamics and evolutionary dynamics have shown possible paths towards information production under template-free elongation [30, 31, 34–36]. However, these studies have not incorporated population structure into their models.

Another important aspect of the transition from pre-life to life is the formation of membranes. Membranes are of interest to the origin of life because they serve many independently useful functions. For instance, they allow for the compartmentalization of reactions. They co-localize elements which could help cooperation and increase reaction rates [37]. Membranes (or other forms of population structure) also help cooperative functions in avoiding the parasitic take-over that occurs rapidly in a well-mixed system [22–24, 26, 38, 39]. Experimental groups have been able to create such compartments, including membrane vesicles that can encapsulate relevant prebiotic polymers and divide [40, 41]. There is also evidence that the building blocks for lipid membranes could have existed in the prebiotic world, either through import by meteorites [42–44], or by reactions in hydrothermal vents [45, 46]. Such molecules are then able to self-assemble into vesicles [47]. It is possible that these vesicles, also called “protocells,” could contain a form of heritable information, either as genetic polymers, or “compositional genomes” [48] and (eventually) the necessary metabolism to maintain its own existence [29, 49].

There has been growing interest in studying the simple vesicle systems that embed genetic polymers in vitro [12, 13, 22, 50–53]. Experimental efforts in this domain are young, and so far, they have often focused on simple template-directed polymerization inside protocells. As finding suitable experimental setups with this many components is difficult and labor-intensive, it is useful to investigate how particular enzymatic activities would affect the dynamics of the system in a theoretical framework. Ganti’s Chemoton model has been a classic linchpin from which many vesicle-polymer models have been built [54–56]. This model, and importantly Eigen’s critical observations on the “error-catastrophe” and models of hypercycles, highlighted key requirements for experimental studies of origin of life [57, 58]. More recent theoretical models of selection within compartments have also provided new insights into their role in promoting replication [24–26, 59, 60]. Some of these insights has been translated successfully to laboratory experiments [22]. Similarly, this study aims to provide a glance into the dynamics that remain difficult to test in the lab, but may be of value in inspiring future experimental studies.

In this vein, we set up a model in which we simulate the dynamics of template-free elongation of polymers within compartments. While template-free production of information has been studied before [30, 34], and membrane dynamics and behavior have also been studied [61–63], an interlocked system in which both of these components play concurrent roles is not well-understood. Nonetheless there is evidence that membranes promote non-enzymatic template-free formation of RNA oligomers during dry-wet cycles [64]. The wetting stage provides fresh building blocks to the system, while the drying concentrates monomers in order to increase the likelihood of bonding. To elucidate the dynamics that are made possible by coupling between membranes and elongating strands, we set up a platform to simulate a system of compartments that contain elongating polymers. Previous approaches such as the Chemoton model and its extensions aim to develop a complete picture of the minimal units of cellular life [54–56]. As such, they come with relatively complex objects like metabolic cycles and template-directed elongation (and replication) as a starting point. Our study is smaller in scope and focused on a simpler objective: we aim to isolate the effects of compartmentalization on patterns within a single type (or a pair) of functional polymer. In our system, genetic polymers can be elongated; however, template-directed replication of these polymers does not occur. We make minimal assumptions about the functionalities that exist in this system. For instance, unlike the Chemoton model, metabolic cycles (and membrane generation) are not considered, and are not coupled with the elongation reactions. Our simplification allows us to better separate the causal effects of compartmentalization.

A key component of our study is the presence of functional subsequences, called *motifs*, that alter the behavior of the compartment they reside in. These motifs are defined as patterns of interest within the sequence, and can be thought of as “schemas” in the context of evolutionary algorithms [65, 66]. Although in that work, in contrast to ours, Darwinian evolution is built into the system. Specifically, we ask how motifs with a simple ability—biasing the type of monomer that is added to the strands inside their compartment—alter the dynamics of the system. Adami and Labar have recently argued that biasing the alphabet in constructing information-carrying molecules can significantly improve the rate at which entropy generates information and perhaps increase the chances of finding replicase polymers [34]. In this study, we use a biasing function and show that such a function can assist (as Adami and Labar claim) in the emergence of interesting and complex behavior. It is noteworthy however, that in modern systems, such biases are not ubiquitous (and replication is template-directed in contrast to our model). Hence, we do our analysis with the understanding that such a bias could have been weakened or removed from the system once enzymatic replication took effect [67].

Biologically, this mechanism could be biasing the membrane import of particular monomers [14–16]. Janas et al. [16] demonstrate the ability of an RNA complex to facilitate the import of tryptophan across a membrane. It is noteworthy that in the case of membrane transport, to the best of our knowledge, there are no known RNA strands that can selectively facilitate the transport of particular monomers. Another possibility is that motifs selectively catalyze the synthesis (i.e. activate monomers or synthesize nucleotides) [17–19] or degradation of their constituent building blocks. We hope that our results would encourage experimentalists to search for and use ribozymes with these abilities in protocell experiments.

Though our work is primarily conceptualized in the context of RNA polynucleotides, our account is consistent with other hypotheses for the earliest information-carrying molecules, such as the coevolution of polynucleotides with polypeptides [68]. We first study the fate of these motifs in compartment systems where only a single motif is active, demonstrating selection in the absence of template-directed replication. We investigate which motif identities thrive under the dynamics they create. Second, we study such motifs in cooperative and competitive dynamics, showing that complex behavior can emerge from the simple mechanism of biasing monomer addition.

## Methods

We simulate a population of compartments containing elongating polymers. The polymers are modeled as binary strands (alphabet ∈ {0, 1} similar to previous studies [31, 32, 65]), and the compartments divide and die according to the Moran process [69]. A binary alphabet is sufficient to encode any larger alphabet size. While simulating larger alphabet sizes directly is possible, binary encoding allows us to keep the model computationally tractable (both for simulation and analysis). Furthermore, it directly resembles the effects of enzymatic bias against a purine or pyrimidine [18]. The strands elongate by attaching an activated 0 or 1 monomer present in the compartment or environment to their terminus (hence the attachment is directed). A single monomer can also be added to an “empty” strand to start a new polymer of length one. The simulation consists of three phases:

**Initiation**: The system starts as a fixed-sized population of *N* empty compartments. Each compartment has a maximum number of possible strands *M*. Hence the total number of strands in the population will never exceed *N* × *M*. Monomer concentrations in the environment remain constant, and the concentration of monomers inside the compartment are equal, unless the compartment is influenced by a motif. After initiation, the system enters the elongation phase.

**Elongation**: During the elongation phase, each strand—including the empty strands—is updated: either it is elongated at its terminus with probability *r*, or it remains unchanged. Strands are capped at some maximum length such that there are no repeating motifs, and for computational efficiency (see S7 Fig for a detailed account of the effects of strand length). The type of the monomer added (0 or 1) is determined with equal probability (*b* = 0.5). However, in the presence of a particular sequence pattern, i.e. a motif (see below), elongation is biased towards a monomer type in its host compartment. Once all strands have been updated, the division phase starts.

**Division**: The division phase works according to the Moran process [69]. A compartment is chosen at random and undergoes division into two. The contents of the parent compartment are randomly distributed between the two daughter compartments. Then, a random compartment from the entire population—including both daughter compartments—is eliminated; hence, the population of compartments remains at the fixed size *N*. At this stage, the process either terminates or re-enters the elongation phase.

**Fig 1:**
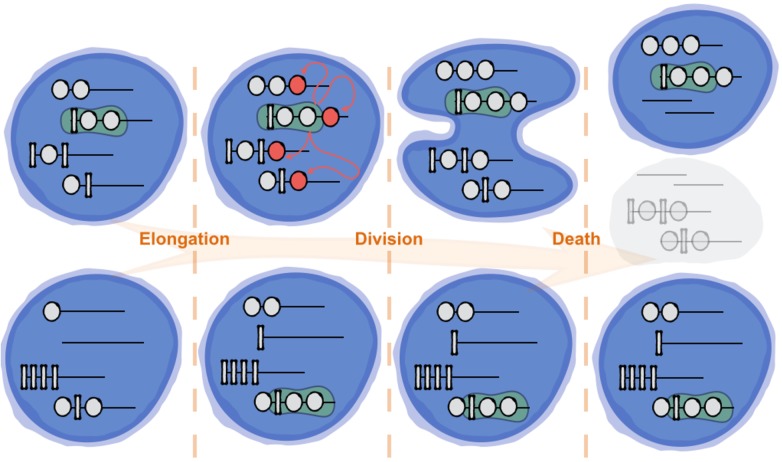
Schematic presentation of the simulation process. The three stages of simulation in each round are shown. Here *N* = 2, *M* = 4, maximum strand length *L* = 7, and the motif is 100. Note that any one of the compartments might be killed after the division event, the choice of a daughter compartment is incidental.

The system undergoes many cycles of elongation and division. In Fig 1, we illustrate the process of elongation and division in a population of size *N* = 2.

**Motifs bias strand content**. As a key component of our model, certain subsequences (e.g. 1001), called motifs, can bias the elongation probability in favor of a specific monomer type. For instance, the presence of the motif 1001 in a compartment may increase the probability of adding a 0 monomer, rather than a 1, to all the sequences within the compartments that contain 1001. We set *b* to be the probability of adding a 0 monomer given an elongation event occurs and the motif is present.

We study the dynamics of the system in two sets of simulations. In the first set, only one pattern is denoted as a motif. Moreover, the presence or absence of a single motif suffices to introduce the bias into a compartment. In the second set, two distinct motifs are used. In this case, the two motifs can bias a compartment towards addition of the same monomer type (which results in a system with similarities to the single motif case), or of opposing types. For the rest of this study, motifs are taken to be of length 5. We present the results for shorter motifs in S8 Fig.

## Results

We simulate the model described in the previous section until the frequency of the motifs in the population reaches steady-state. We then investigate the effects of the motif on the resulting steady state. Our results are presented in two sections. First, we examine the fate of a population when there is only one motif pattern. In this case, whenever the compartment contains one or more resident motifs, the elongation is biased. Second, we expand the repertoire of motifs to two, using the complements of those studied in the single motif case. In the two motif case, the more frequent motif dictates the direction and intensity of bias introduced in the compartment.

In what follows, we present the results for a fixed set of parameters (*L* = 7, *N* = 100, *M* = 100, *r* = 0.05, and motif size 5), and varying bias *b*. These parameters were chosen such that enough trials can be run for computing confidence intervals in reasonable time. Increasing *M*, *L*, and *N* do not change the behavior that we are interested in qualitatively. Smaller values are less plausible biologically, but do not have strong effects either. We show their effects in S5–S7 Figs. Varying *r* has more complicated effects on the frequencies of the motifs, opposing effects in cooperative vs. competitive dynamics (S2 and S3 Figs), for reasons that will become clear.

### Motif frequency is affected by the motif composition and pattern

**Fig 2:**
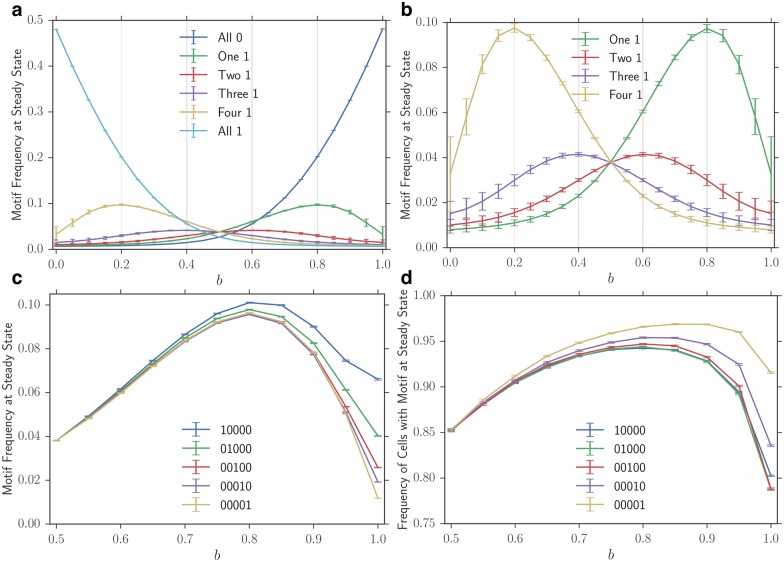
Effect of motif identity on steady-state motif frequency. **(a)** The effect of motif composition on the steady-state motif frequency. Each data sequence label refers to the number of 1 and 0 monomers in the motif of length 5; e.g., ‘One 1’ is the average of steady-state motif frequency for simulations using 10000, 01000, 00100, 00010, and 00001 motifs. All possible motifs of length 5 are examined. **(b)** The same data displayed except for the ‘All 0’ and ‘All 1’ strands to better illustrate the contrast between patterned strands. **(c)** The effect of motif pattern on the steady state motif frequency for length 5 motifs with a single 1 monomer. **(d)** The effect of motif pattern on the frequency of compartments with motifs at steady state for this same set of motifs. Data sequences are the means of 50 trials of steady-state motif frequencies with 95% confidence intervals. All simulations are run with *r* = 0.05, *N* = 100, *M* = 100, and a maximum strand length of 7.

We first explore the effect of motif composition, i.e. the relative number of 0 and 1 monomers in the motif strand by examining motifs of length 5. Intuitively, one would expect motifs with a higher ratio of the monomer which they bias their compartment to should perform better. This is indeed the case. In fact, we observe that the motif frequency at steady state is maximized near the bias which corresponds to the ratio of the monomer that is favored (Fig 2a and 2b). In the most obvious case, when the motif is entirely made up of 0 monomers, i.e. 00000, the motif frequency is maximized when *b* = 1.0, meaning that when the motif is present, all added monomers will be 0 monomers (Fig 2a). One can think of this motif as the most elementary template-free replicase.

The results become more interesting when we look at motifs that have the same relative ratio of monomers but do not share the same patterns. Consider the motifs of length five with four 0 monomers and one 1 monomer, i.e. 10000, 01000, 00100, 00010, and 00001 (Fig 2c and 2d). When *b* = 0.5, there is no difference in the steady-state frequency of motifs, regardless of their particular pattern. However, as the bias *b* approaches 1.0, we observe that motifs with the 1 monomer towards the beginning (e.g. 10000) achieve higher frequencies at steady state relative to motifs with the 1 monomer in final positions (e.g. 00001). Under higher biases, the number of compartments containing a 10000 motif is lower, while the less frequent 00001 motifs are present in more compartments, albeit at a lower abundance per compartment (Fig 2d).

**Fig 3:**
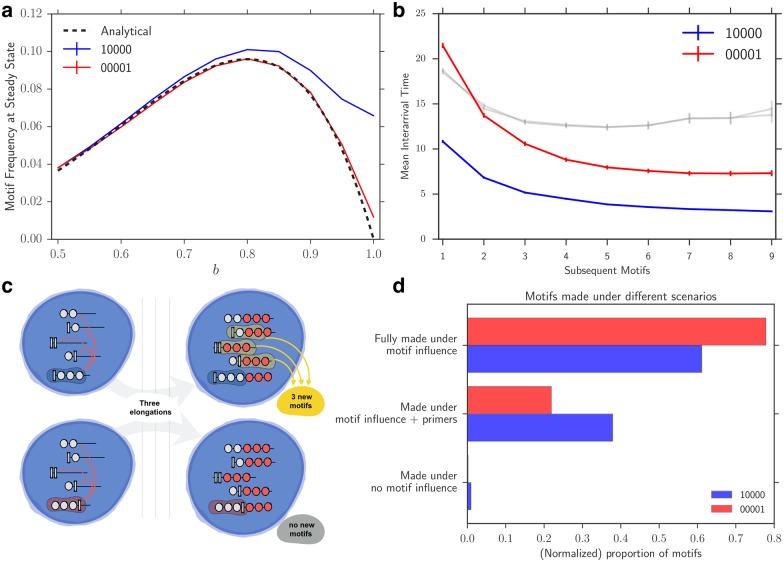
The role of primers in breaking symmetry. **(a)** The motif frequency at steady state for 10000 (blue) and 00001 (red) shown along with an analytical approximation (black dashed line) that ignores primers (See S1 File for details). **(b)** To observe the influence of each motif on the speed of production of its copies we measure the inter-arrival time of motif appearances since the first motif emerges. The grey lines show that there is no difference when *b* = 0.5, however 10000 has significantly lower inter-arrival times when bias is high (shown with *b* = 0.9 and *r* = 0.05) **(c)** An illustration of how 1000 benefits from primers. Two compartments with the motifs of interest are shown, where they contain all possible primers of length 2. In the limiting regime where *b* = 1.0 and elongation probability *r* = 1, the 1000 motif is generated by 3 out of the 4 primers after three steps (top), whereas 0001 is not generated at all (bottom). **(d)** This speedup can be ascribed to the fact that a larger portion of 10000s are made out of primers, and hence require fewer rounds to result in a complete motif. This is shown with *b* = 0.9 and *r* = 0.05.

To understand this discrepancy between the steady-state frequency of mirrored patterns, we compare how 10000 and 00001 affect the dynamics of the population. We consider these two motifs because they are especially good examples of this effect, diverging more clearly in their steady-state frequencies. We depict this divergence in Fig 3a along with an analytical approximation that is discussed in the S1 File.

We observe that once the first motif appears in a neutral compartment, the subsequent 10000 motifs are produced more quickly relative to their mirrored motif 00001 (Fig 3b). Namely, the 10000 requires fewer rounds to influence the production of copies. We also observe that when the ratio of elongation to division in the simulations increases, this effect is weakened (see S2 Fig). This suggests that if the 00001 is given more opportunities to influence more elongations before division, this symmetry breaking is less significant.

We also record whether each monomer is added to its polymer in the presence (or absence) of a motif. In Fig 3c, we show that 00001 motifs are often produced entirely in the presence of themselves. In contrast, a significant fraction of 10000 motifs build off of existing precursor strands, “primers,” that are created without a motif-containing strand in the same compartment (see a detailed account in S2 Fig). These two pieces of empirical information, as well as the close match between the approximation that ignores primers with the frequency of 00001, make it clear that the advantage for 10000 motifs is due to their reliance on primers. Specifically, if the elongation probability is high, motifs that use primers need less time to produce copies of themselves. To elucidate this point, consider the limiting case where a motif with strong bias (*b* = 1.0) is introduced in a typical compartment. In this case a 10000 motif will produce copies from a large proportion of the existing strands, i.e. 1/2 of the strands of length 1, 3/4 of length 2 polymers, 7/8 of polymers of length 3, 7/16 of the length 4 strands, and so on. This also explains the high density of 10000 motifs within the compartments in which they exist. A simple example is illustrated in Fig 3c. However, this compartment will also include a considerable number of all-0 polymers. On the other hand, a 00001 motif with *b* = 1.0 is unable to directly produce any copies of itself, resulting in very low concentration of such polymers in motif-containing compartments. There will be a large number of all-0 polymers that are only able to generate the motif when they have separated from the 00001-containing compartment.

### Motif sequences affect their ability to cooperate with their complements

In the simulations above we observed two phenomena. First, motif frequency is maximized around the bias parameter *b* that corresponds to the ratio of zeros and ones in the motif. Second, fixing the monomer ratio, the order at which the monomers are arranged is important in the steady state frequency of the motif. In this section, we study the dynamics of the system with pairs of complementary motifs. Given our previous observations, a motif is called cooperative if it biases the system towards the composition of its complement, whereas it would be considered competitive if it biases the system towards its own composition. The cooperative case is particularly interesting because it starts to resemble template-directed elongation.

To build on the single motif case, we consider the patterns 10000 and 00001 that showed the most divergent steady-state frequencies. We expand the repertoire by adding their complements in the system as motifs. For instance, in the system with the 10000 motif, now 01111 would also be a motif. The dynamics are considered cooperative if 10000 biases the compartment towards 1s and 01111 biases the compartment towards 0s. Likewise, it would be considered competitive if 10000 biases the compartment towards 0s and 01111 biases the compartments towards 1s. For simplicity, we assume that the intensity of bias is similar for the two motifs, i.e. both bias the compartment by the same amount, and if there is equal numbers of two motifs in the compartment, the compartment will behave as neutral in both dynamics (for instance the complements may form double-strands and lose the ability to fold).

Recall that in the single motif case, 10000 performed better than its mirror pattern 00001. For competitive motif pairs, we observe that 10000 and its complement have a higher steady state frequency than 00001 and its complement. However, the motifs and their complements perform similarly with respect to each other and the sum of their frequencies is similar to the total frequency of the 10000 motif in the single motif case (when bias is strong). Essentially, we observe that the population of compartments is split into two groups, each primarily under the influence of one of the motifs.

**Fig 4:**
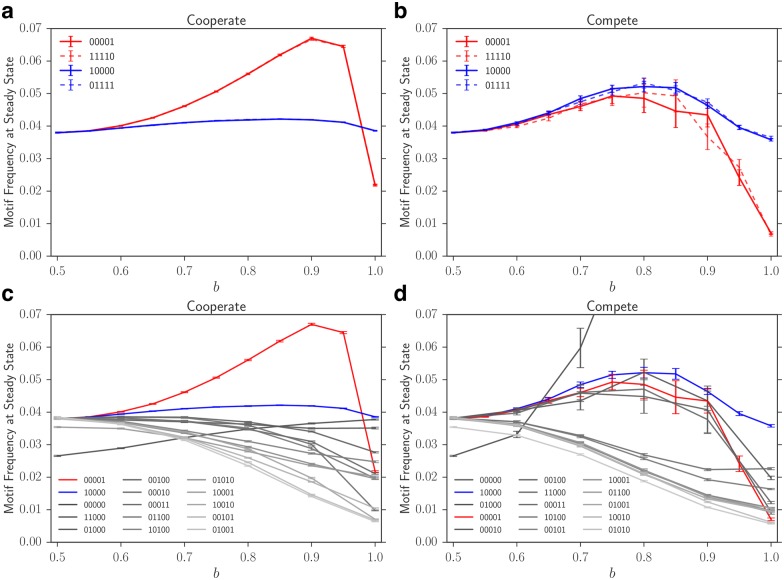
Steady state motif frequencies in cooperative and competitive dynamics. Steady state frequency of motif-containing strands as a function of the bias in cooperative and competitive dynamics. In all cases, *r* = 0.05. Motif steady state distributions are the average of 50 trials. The values of *b* are shown for the motif that promotes the creation of 0 monomers (11110 and 01111 in subfigures (a) and (c), 00001 and 10000 in subfigures (b) and (d)). The other strands in the same subfigure promote 1 monomers with probability 1 − *b*. Shown are steady state motif frequencies for **(a)** The pairs A: 11110 and B: 00001 (in red) and A: 01111 and B: 10000 (in blue) under cooperation, **(b)** The pairs A: 11110 and B: 00001 (in red) and A: 01111 and B: 10000 (in blue) under competition, **(c)** all possible mirrored motif pairs of length 5 in the cooperating case (only one of the pair is shown since the two strands that make up the pair were indistinguishable from each other on average), and **(d)** all possible mirrored motif pairs of length 5 in the competition case. Note that in **(d)**, the pair A: 00000 and B: 11111 grows much faster in comparison to the other pairs, and its behavior at high bias is not shown in order to make differences between other pairs more clear.

**Fig 5:**
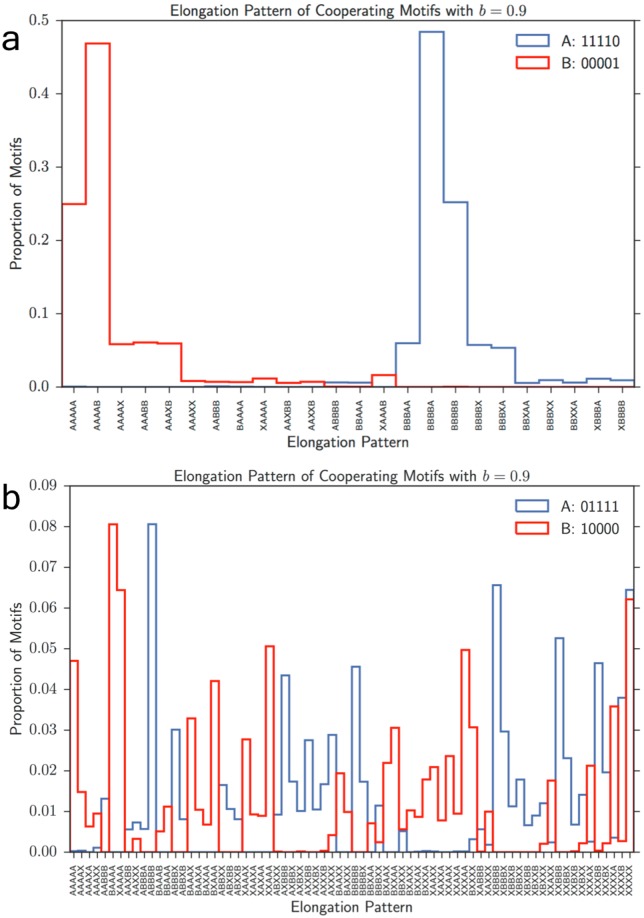
Elongation under the influence of cooperative motifs. **(a)** Motif pair *A*: 11110 and *B*: 00001 successfully cooperate by building each other’s precursors. The values on the x-axis show whether the particular monomer was added under the influence of motif *A*, motif *B*, or neither (denoted by *X*). **(b)** Same plot for motif pair *A*: 01111 and *B*: 10000, shows that such cooperative relationship does not exist for this pair, hence they do not exhibit selective advantage through biasing the monomer balance towards the main element of their partner. *N* = 100, *M* = 100, *r* = 0.05, and maximum strand length 7 for all runs. Only patterns above a frequency of 0.005 are shown. Average of 15 trials.

Interestingly, the pattern 00001 which lost to 10000 in both the single and double motif cases, achieves the highest steady-state frequency across all paired simulations when cooperating. The total frequency of the motif and its complement also exceed the steady-state frequency of all the patterns (barring 00000) in the single motif simulation. These observations are presented in Fig 4. This happens because active (motif-driven) production of sequences under high biases results in larger fraction of primers that are conducive to producing motifs of the 00001 type. Additionally, note that for a significant fraction of the cooperative 00001 motifs, the entirety of the sequence is generated under favorable bias (see Fig 5a). I.e. the monomer being added to the strand is the more likely type under the bias. Recall that in the competitive case (both as pairs, and as single motifs) this is not true: many primers are built under no bias, or a large fraction of monomers have to be added while the bias is unfavorable. This reduces the total frequency of the desired motif.

We now investigate the dynamics of the two-motif simulations to understand the behavior we observe in Fig 4. We revisit how the motifs are made; in particular, whether a motif is built in presence or absence of itself, its cooperator/competitor, or none. In Fig 5a, we show that for the cooperating pair *A*: 00001, *B*: 11110, most motifs are built under the following process: the dominating cooperator (the motif with higher frequency in the compartment) builds the first four pieces of the other sequence, namely its primer. For instance *A* builds 1111 and *B* builds 0000. Then as soon as the dominant cooperator is changed, it will first use the primer to make a large number of self copies. Namely, if *B* is initially dominant and makes many 0000 primers, once *A* becomes majority (by chance or division), then the first step will entail adding 1s to the primers and producing many 00001s. These dominating *A*s will then start making 1111 which will serve as a primer for *B*. The cycle, an example of a “hypercycle” [58], completes by *B* re-emerging as the dominant motif, making self-copies, and beginning to make primers for *A*.

These results are summarized in Fig 5a, and in contrast, one can see that the other pair *A*: 10000, *B*: 01111 do not show this cooperative behavior (Fig 5b). In this case, if *B* were dominant, it would immediately start creating *A* strands by elongating existing primer strands that were not generated under motif influence. This continues until *A* becomes dominant. Then *A* immediately begins creating *B* strands and fails to create the self copies or primers observed in previous case. Hence these strands cannot cooperate as efficiently.

These observations are further supported by noting that increasing the rate of elongation relative to division causes the successful pair to do increasingly better when cooperating (S3 Fig). As more elongation occurs, the pair is able to climb to higher frequencies by continually oscillating between which motif is dominant. 10000 and its complement do not have large jumps in frequency. Thus the 10000 pair is disadvantaged by this increase in elongation rate. Recall that the effect of elongation rate in the single motif case was the opposite: Increasing the elongation rate reduced the difference between 10000 and 00001 (in that case 10000 did better) as seen in S2 Fig. There, increasing the elongation rate reduced the advantage provided by neutrally made primers, as more elongations would occur before a compartment division. Here, more elongations per division boosts the cooperative process, as precursors are actively made.

## Discussion

Through this simulation study, we make three main observations:

1. *Template-free elongation can undergo selection*. It has been suggested before that information stored in some form in the environment (e.g. inherent biases in the chemical production) can result in selection of sequences [10, 31, 32]. Here we show an explicit case of such selection arising from an elementary process that is not dependent on template-directed replication. Bias in the motif production could in turn result in structures that are capable of template-directed ligation and other useful functions [67]. Though primarily grounded in an RNA world, our results may equally apply to any system of polymers with similar chemical functions, such as oligopeptides (the caveat is that elongation needs to be directional). For a related example where peptides are considered as channel precursors (leading to similar chemical behavior as our motifs), see Ruiz-Mirazo and Mavelli’s simulation of lipid-peptide cell [14].
2. *Dividing compartments result in surprising asymmetries in motif selection*. We observed that as a result of elongation occurring inside a dividing compartment (and changing local environment associated with that), patterns that are mirrored perform differently. Further investigation showed that this arises because certain motifs are better at utilizing precursors, and therefore require fewer elongations on average. Thus, motifs that are produced quickly (with the aid of primers) enough under the biased environment before the division of the compartment gain an advantage over motifs that are built from scratch every time. Note that the presence of this kind of population structure (where local environments form), coupled with occasional divisions is necessary for these effects to manifest themselves.
3. *Elongation bias can result in cooperative dynamics, which in turn yields selective advantage to cooperators*. Finally, we observe that in systems with more than one motif, interesting cooperative and competitive dynamics can emerge. Even though we only assume one possible function in our system, we observe a drastic difference in the performance of sequences that appear similar (symmetric in composition), depending on whether they are cooperating or competing. These results highlight that cooperative functions can arise from very simple and prebiotically plausible rules.

As this is a simulation study, we have had to make a number of simplifying assumptions to capture the essential effects in the system, while keeping the number of parameters small and the model computationally tractable. Making such choices introduces some limitations to our study’s generalizability. We mention some of these limitations in what follows.

In some studies of membrane dynamics, including work by two authors of this manuscript [26], it has been argued that a “division into many” scenario may be possible and more helpful to enzymatic molecules than a binary division [63]. However, studies have also demonstrated processes in which compartments or droplets undergo binary division [61, 62]. We have chosen the latter approach for simplicity and generalizability, as from this study’s perspective division into many is an extension of binary division (with smaller compartments). In our case, it allows us to replicate the Moran process, which is well-understood from a theoretical perspective.

In our model we only explicitly address the case where the effects of the motif only depend on its presence (or absence) in the compartment. Accordingly, in the paired motif cases, the motif’s effect is determined by the motif that is simply more abundant in the compartment. We recognize that this is the most simplistic approach. A more realistic approach would map the level of bias introduced in the compartment to the concentration of motifs within the compartment. This is a possibility for extending our model.

Another potential limitation arises from the synchronization between elongation or degradation of strands with the division or death of compartments. This manifests itself in S2 Fig. where selection is reduced as *r* grows (relative to *N* and *M*) in the single motif case. But at the same time, an increase in *r* also improves cooperation effects (see S3 Fig). Hence in both parameter regimes our study provides interesting effects that may be harnessed in a compartmentalized population.

Finally, as we mentioned before, other extensions of our model would be to expand the sequences space using larger alphabet sizes (in particular 4 and 20), as well as discontinuous motifs, as observed in modern biological systems. The number of possible sequences to analyze is much larger in those cases, and hence drawing a complete picture is more challenging than in the present study.

Nonetheless, we do not expect any of these assumptions to affect the three main conclusions highlighted above. While changing the alphabet size may affect how a particular motif may perform compared to another, the principle that some sequences may benefit from primers (or cooperation) more than others remains unchanged. This effect primarily arises because of the directionality of sequence elongation, coupled with cycles of motif influence (or lack thereof) within each protocell.

The question of how complexity and cellular life arose on early earth remains unanswered. As we discussed in the introduction, prominent experimentalists within the field have over the years attempted to make efficient RNA polymerases [8, 9, 20, 70]. The premise behind this approach is that once there is an RNA replicase, it will kickstart Darwinian evolution and hence allow for mutations and natural selection to generate complexity in the familiar way. This approach suggests that the emergence of an early RNA replicase would be the fundamental invention that laid the very foundation of cellular life. An alternative scenario is that replication was latent in the origin of life. Namely, primitive cells (which are not as crucial in the replicase-first case) would originally host a multitude of weak functions that in collaboration with each other could lay the foundation for information transfer across vesicles [71, 72]. Our proposed mechanism in this study has useful insights for the proponents of both scenarios. This study provides a clear example of how a simple function, which is not replication, shows some of the benefits of replicating systems. Moreover, our agents are in essence local manipulators of building blocks, a weak and simple function that we show can produce interesting behavior. This type of function can potentially be employed in concert with multiple other functions (e.g. division inducers, ligases, etc.) to produce complex cells that may eventually be able to behave similar to modern systems. While a simple bias inducer function may not be known in current the repertoire of ribozymes (just like the generic RNA-polymerase itself), given what it can achieve as we show in our simulations, we argue that it is worth looking for.

## Supporting information

S1 Fig The steady state distribution of motifs is robust to initial conditions and stable during simulations. **(a)** The steady-state distribution of the system, reached after about 600 time steps, is insensitive to initial conditions. The darker lines indicate two examples of trials with the same initial conditions that were used for the analysis. **(b)** The steady-state reached after 600 steps is stable for larger time scales (20 trials shown, one in darker color). The red box indicates the region (1000, 7000) that was sampled for the analysis elsewhere in this study.
(TIF)

S2 Fig Elongation pattern. **(a)** Effect of elongation probability on the ratio of the two motif frequencies at steady state. Increased elongation rates relative to division reduces the advantage of the 10000 motif in producing copies quickly. **(b)** This figure is a more detailed presentation of Fig 3d. Histogram shows the proportion of motifs that have each specific elongation pattern. A ‘+’ indicates that a particular monomer was added when a motif was present in the same compartment. A ‘-’ indicates the absence of a motif. The plot ranges from the motif being created entirely without the presence of a motif (− − − − −) to being created entirely in the presence of a motif (+ + + + +). The graphs are for *b* = 0.9 and *r* = 0.05 averaged over 12 trials. Blue represents the 10000 motif and red represents 00001 motif. Only patterns with a frequency above 0.005 are shown. *N* = *M* = 100 and maximum strand length is 7 for all shown simulations.
(TIF)

S3 Fig Effect of elongation rate on success of cooperators. Effect of elongation rate on the ratio of the two motif frequencies at steady state under cooperative dynamics. *N* = *M* = 100 and maximum strand length is 7 for all shown simulations. Motif frequencies were computed by taking the average of 50 trials, after which we computed the ratio.
(TIF)

S4 Fig Elongation under the influence of competing motifs. **(a)** Motif pair *A*: 00001 and *B*: 11110 compete with each other, existing only when they create themselves in entirety. The values on the x-axis show whether the particular monomer was added under the influence of motif *A*, motif *B*, or neither (denoted by *X*). **(b)** Same plot for motif pair *A*: 10000 and *B*: 01111, shows that these competitors are able to create themselves from precursors. *N* = 100, *M* = 100, *r* = 0.05, and maximum strand length 7 for all runs. Only patterns above a frequency of 0.005 are shown. Average of 15 trials.
(TIF)

S5 Fig Effects of changing M on the steady state frequency of motifs. Larger capacity for strands inside the compartment results in a smaller difference between the motif frequencies. This is expected as a larger compartment is increasingly more like a non-compartmentalized system, where this difference is not expected. These simulations done with *L* = 7, *N* = 100, *r* = 0.05. Dotted lines denote the analytical approximation. More strands per cell increase the likelihood that a cell contains a motif and decreases the time to obtain a motif, resulting in more strands elongated under bias. This explain a higher frequency of motifs. Note that for smaller *M*, a larger proportion of generated motifs are produced under no bias. Because the approximation does not account for neutrally made motifs, for small *M* (where the motif arrives relatively late) it underestimates the frequency of motifs in the cell.
(TIF)

S6 Fig Effects of changing N on the steady state frequency of motifs. Compartment population does not affect the advantage of the primed motifs. These other parameters in these simulations are *M* = 100, *r* = 0.05, *L* = 7. Dotted lines denote the analytical approximation. Larger populations result in more elongations per death, hence longer sequences on average, which results in higher total frequency of motifs.
(TIF)

S7 Fig Effects of changing the maximum strand length. Larger maximum strand length preserves the qualitative advantage of primed motifs over non-primed ones in high biases. The limiting behavior depends on *r*, the rate of elongation. These simulations done with *M* = 100, *N* = 100, *r* = 0.05. As expected, longer strands result in more opportunities for motifs to arise.
(TIF)

S8 Fig Effects of changing motif length. Smaller motifs are more abundant. The asymmetry in abundance between primed and non-primed motifs is preserved (for high biases). Dotted lines denote the approximation. These simulations are performed with *M* = 100, *N* = 100, *r* = 0.05, *L* = 7.
(TIF)

S9 Fig Numerical verification of Eq 3. Lines represent the approximation for the probability that a strand contains a motif (Eq 3. in S1 File). To compute the probability of a motif per strand numerically, we generate all possible strands up to length *L*, count the number of motifs present and subsequently weight those by the probability that a strand reaches a particular length. Dots show the results for this computation. We show various maximum strand lengths and four different motifs with various patterns of overlaps: **(a)** 10000, **(b)** 10101, **(c)** 101, **(d)** 11001. Strand length distribution was calculated using *r* = 0.05, *N* = 100.
(TIFF)

S1 File Details of simulations and analytical approximations. We provide additional details for the simulations and the robustness of our results. We also provide an analytical approximation for the steady-state frequency of motifs.
(PDF)
