# Supplementary figures and images for: Prebiotic selection for motifs in a model of template-free elongation of polymers within compartments
Source: PLoS One. 2017 Jul 19;12(7):e0180208. doi: 10.1371/journal.pone.0180208 (PMC5516967; doi:10.1371/journal.pone.0180208)

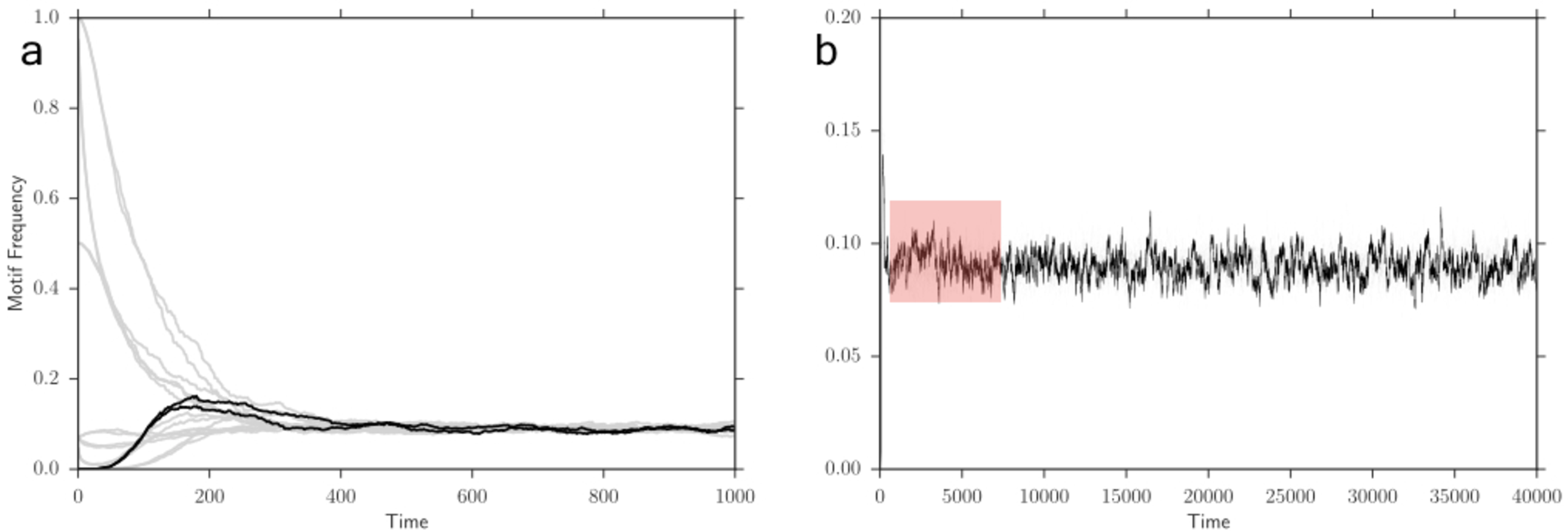

Supplement: S1 Fig — (a) The steady-state distribution of the system, reached after about 600 time steps, is insensitive to initial conditions. The darker lines indicate two examples of trials with the same initial conditions that were used for the analysis. (b) The steady-state reached after 600 steps is stable for larger time scales (20 trials shown, one in darker color). The red box indicates the region (1000, 7000) that was sampled for the analysis elsewhere in this study. (TIF) [file pone.0180208.s001.tif]

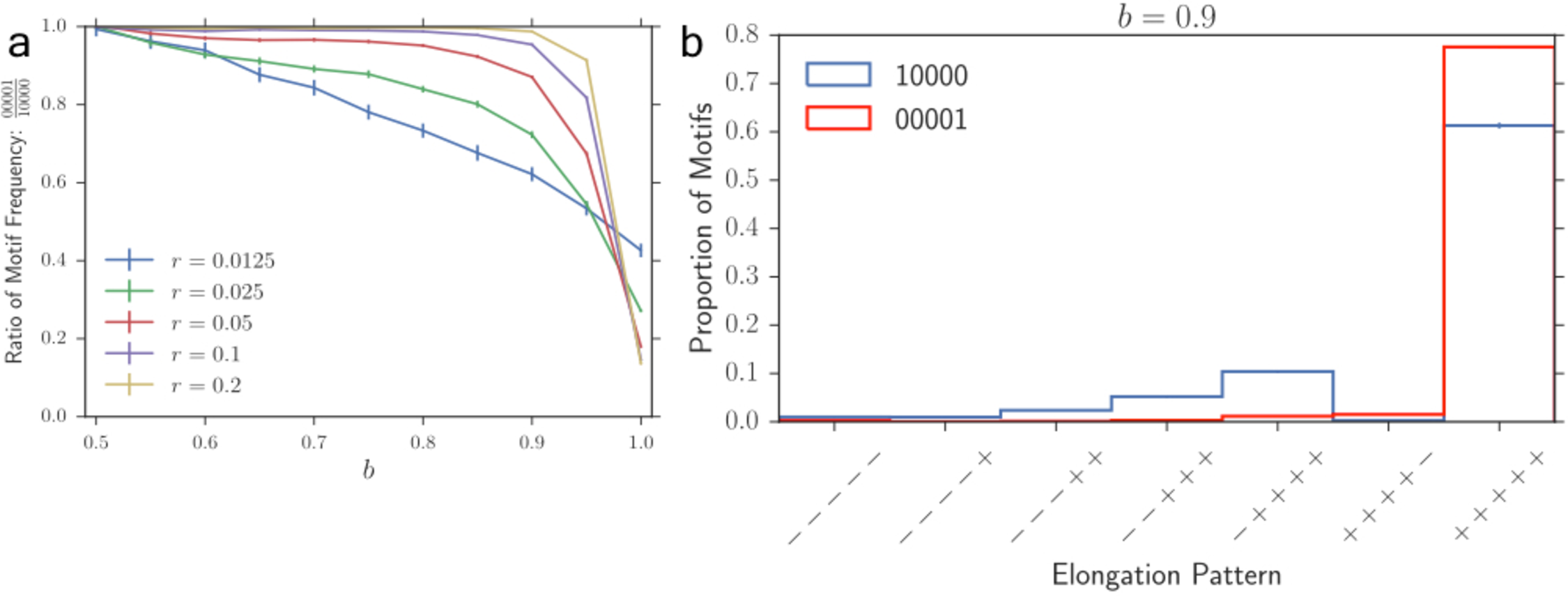

Supplement: S2 Fig — (a) Effect of elongation probability on the ratio of the two motif frequencies at steady state. Increased elongation rates relative to division reduces the advantage of the 10000 motif in producing copies quickly. (b) This figure is a more detailed presentation of Fig 3d. Histogram shows the proportion of motifs that have each specific elongation pattern. A ‘+’ indicates that a particular monomer was added when a motif was present in the same compartment. A ‘-’ indicates the absence of a motif. The plot ranges from the motif being created entirely without the presence of a motif (− − − − −) to being created entirely in the presence of a motif (+ + + + +). The graphs are for b = 0.9 and r = 0.05 averaged over 12 trials. Blue represents the 10000 motif and red represents 00001 motif. Only patterns with a frequency above 0.005 are shown. N = M = 100 and maximum strand length is 7 for all shown simulations. (TIF) [file pone.0180208.s002.tif]

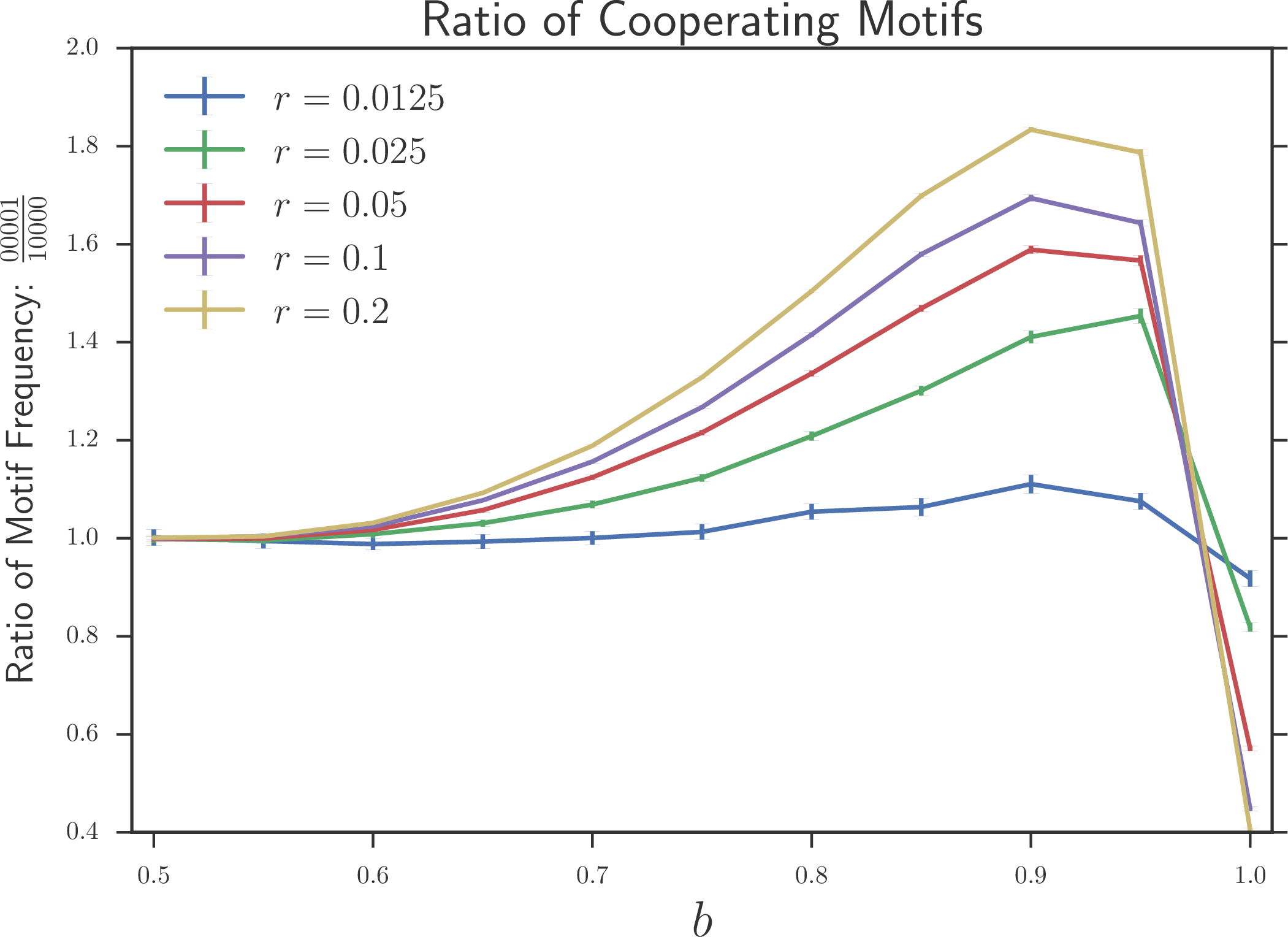

Supplement: S3 Fig — Effect of elongation rate on the ratio of the two motif frequencies at steady state under cooperative dynamics. N = M = 100 and maximum strand length is 7 for all shown simulations. Motif frequencies were computed by taking the average of 50 trials, after which we computed the ratio. (TIF) [file pone.0180208.s003.tif]

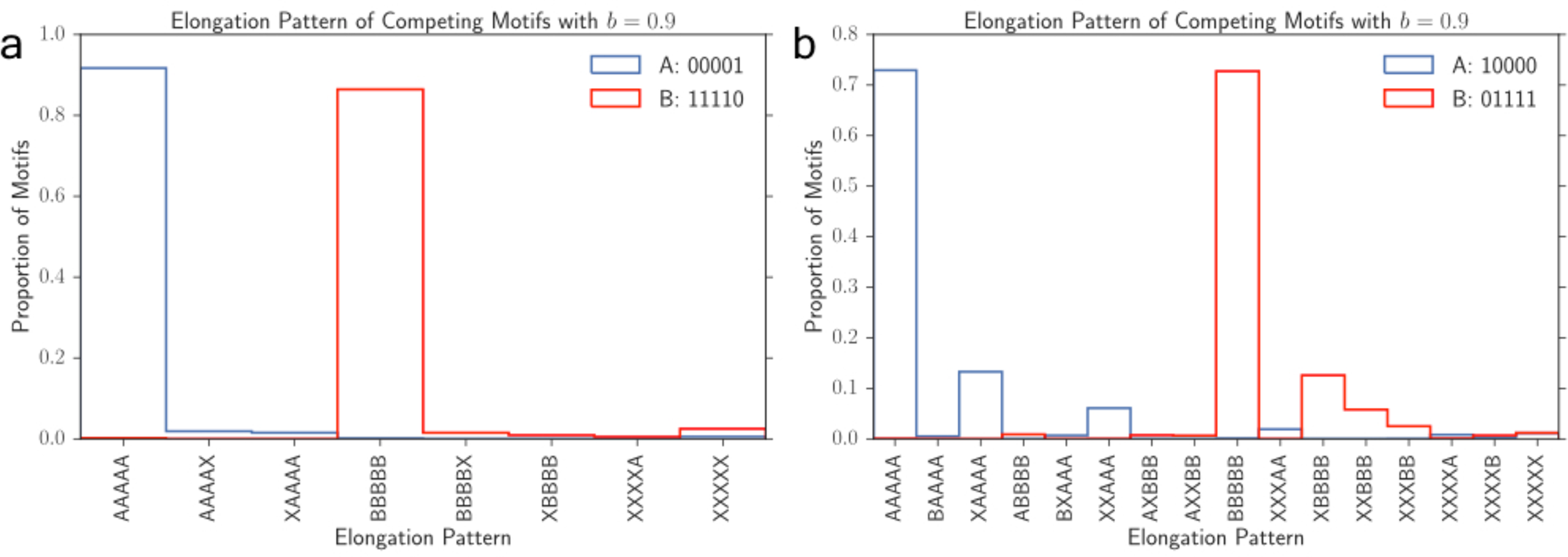

Supplement: S4 Fig — (a) Motif pair A: 00001 and B: 11110 compete with each other, existing only when they create themselves in entirety. The values on the x-axis show whether the particular monomer was added under the influence of motif A, motif B, or neither (denoted by X). (b) Same plot for motif pair A: 10000 and B: 01111, shows that these competitors are able to create themselves from precursors. N = 100, M = 100, r = 0.05, and maximum strand length 7 for all runs. Only patterns above a frequency of 0.005 are shown. Average of 15 trials. (TIF) [file pone.0180208.s004.tif]

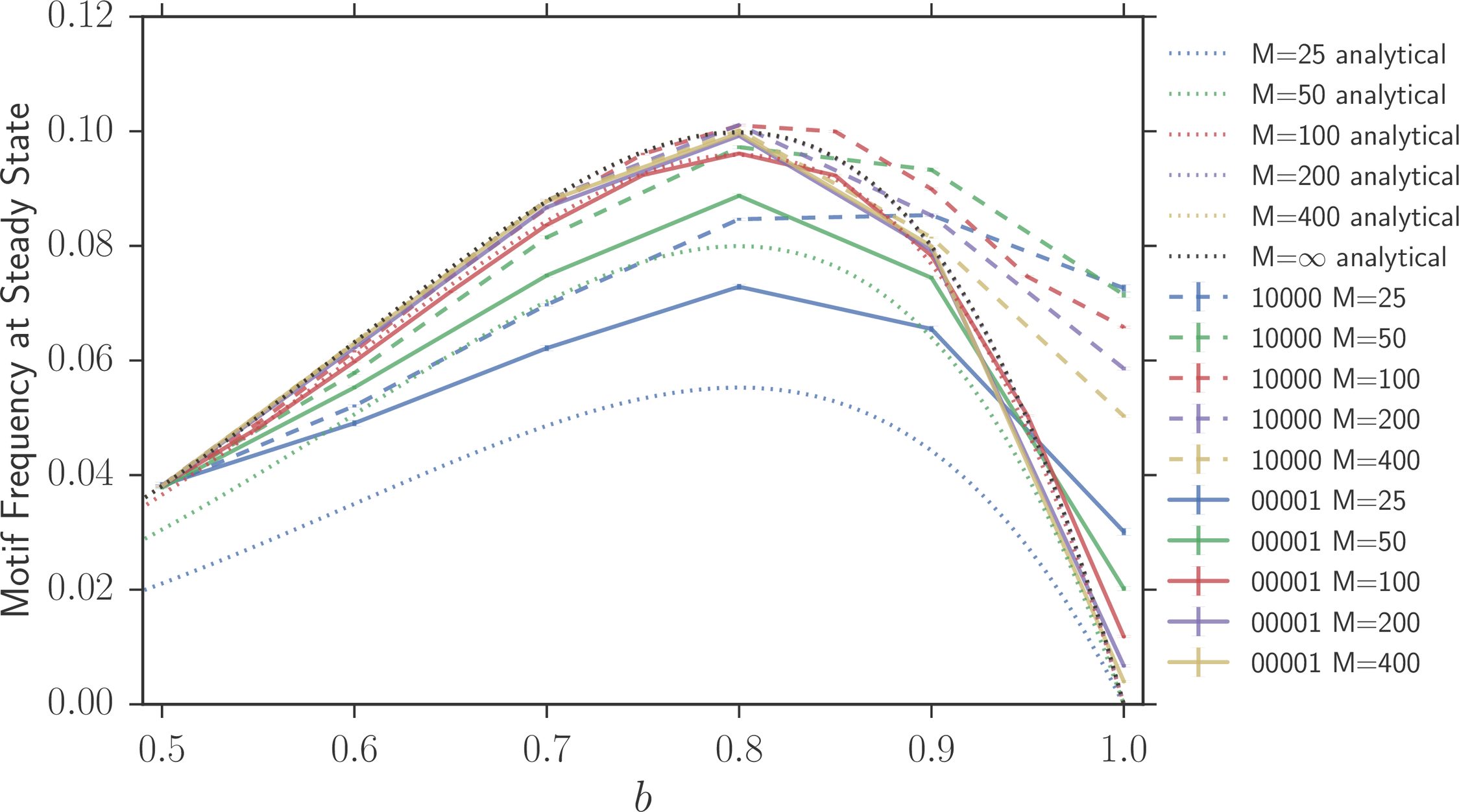

Supplement: S5 Fig — Larger capacity for strands inside the compartment results in a smaller difference between the motif frequencies. This is expected as a larger compartment is increasingly more like a non-compartmentalized system, where this difference is not expected. These simulations done with L = 7, N = 100, r = 0.05. Dotted lines denote the analytical approximation. More strands per cell increase the likelihood that a cell contains a motif and decreases the time to obtain a motif, resulting in more strands elongated under bias. This explain a higher frequency of motifs. Note that for smaller M, a larger proportion of generated motifs are produced under no bias. Because the approximation does not account for neutrally made motifs, for small M (where the motif arrives relatively late) it underestimates the frequency of motifs in the cell. (TIF) [file pone.0180208.s005.tif]

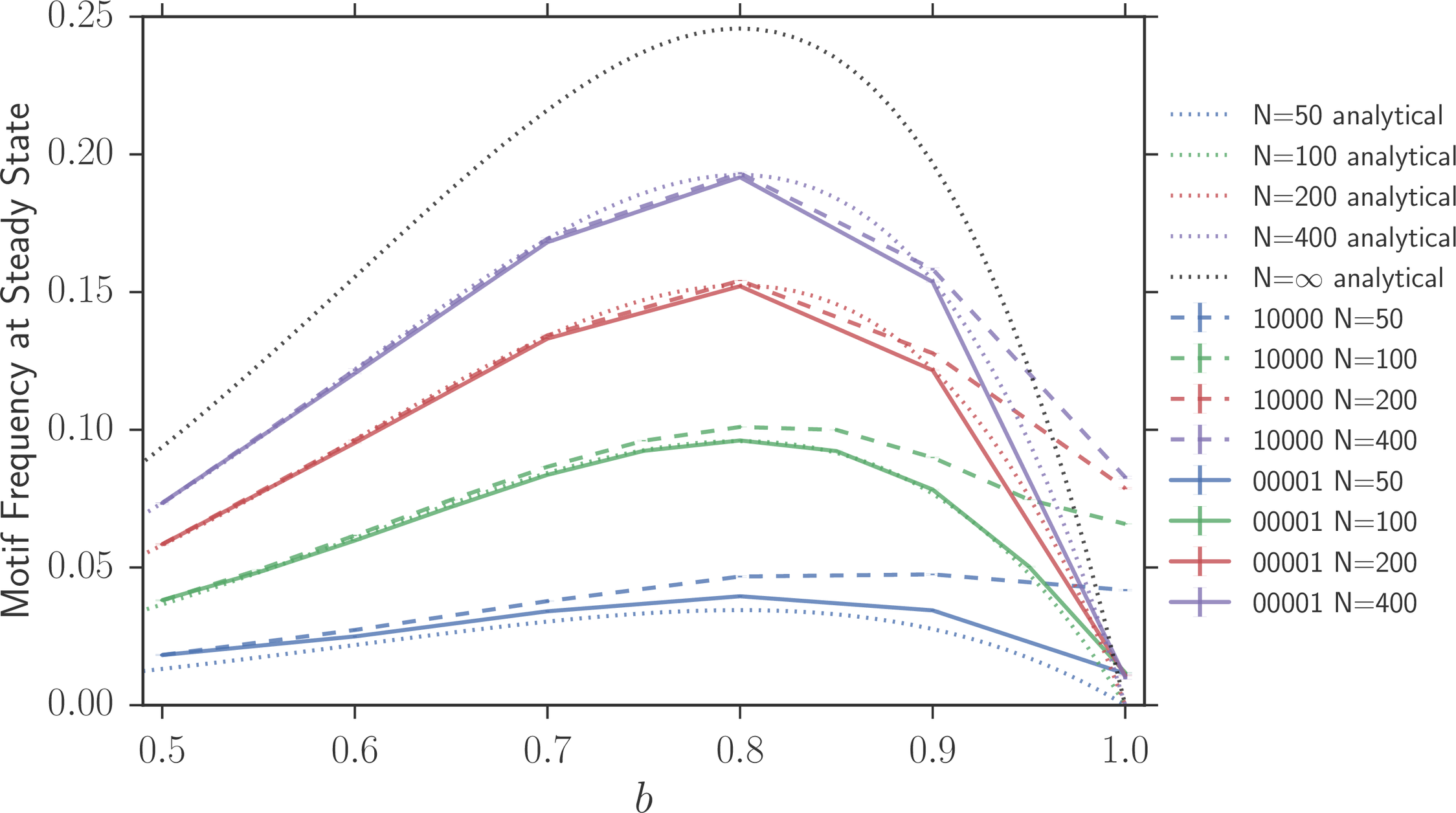

Supplement: S6 Fig — Compartment population does not affect the advantage of the primed motifs. These other parameters in these simulations are M = 100, r = 0.05, L = 7. Dotted lines denote the analytical approximation. Larger populations result in more elongations per death, hence longer sequences on average, which results in higher total frequency of motifs. (TIF) [file pone.0180208.s006.tif]

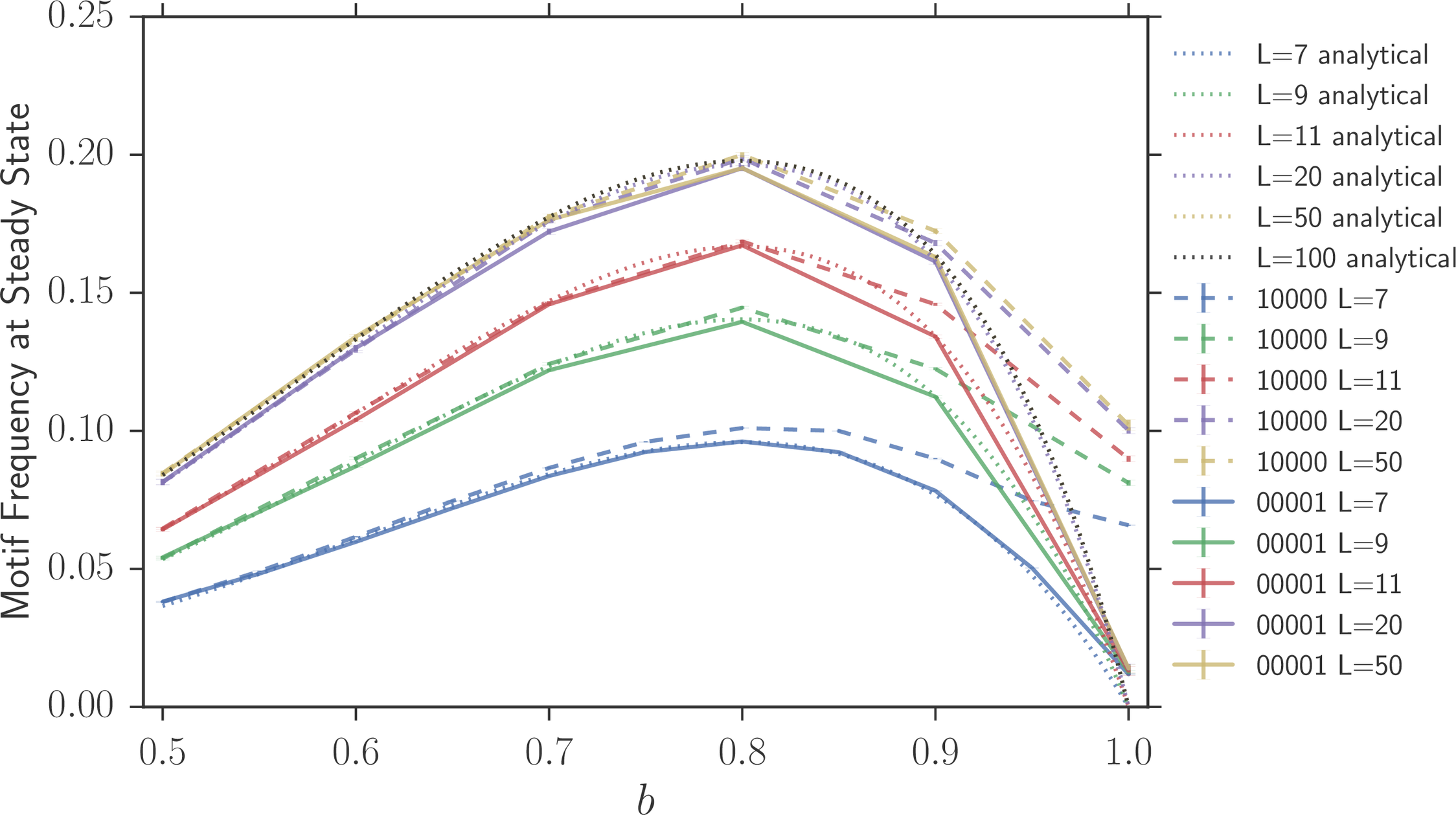

Supplement: S7 Fig — Larger maximum strand length preserves the qualitative advantage of primed motifs over non-primed ones in high biases. The limiting behavior depends on r, the rate of elongation. These simulations done with M = 100, N = 100, r = 0.05. As expected, longer strands result in more opportunities for motifs to arise. (TIF) [file pone.0180208.s007.tif]

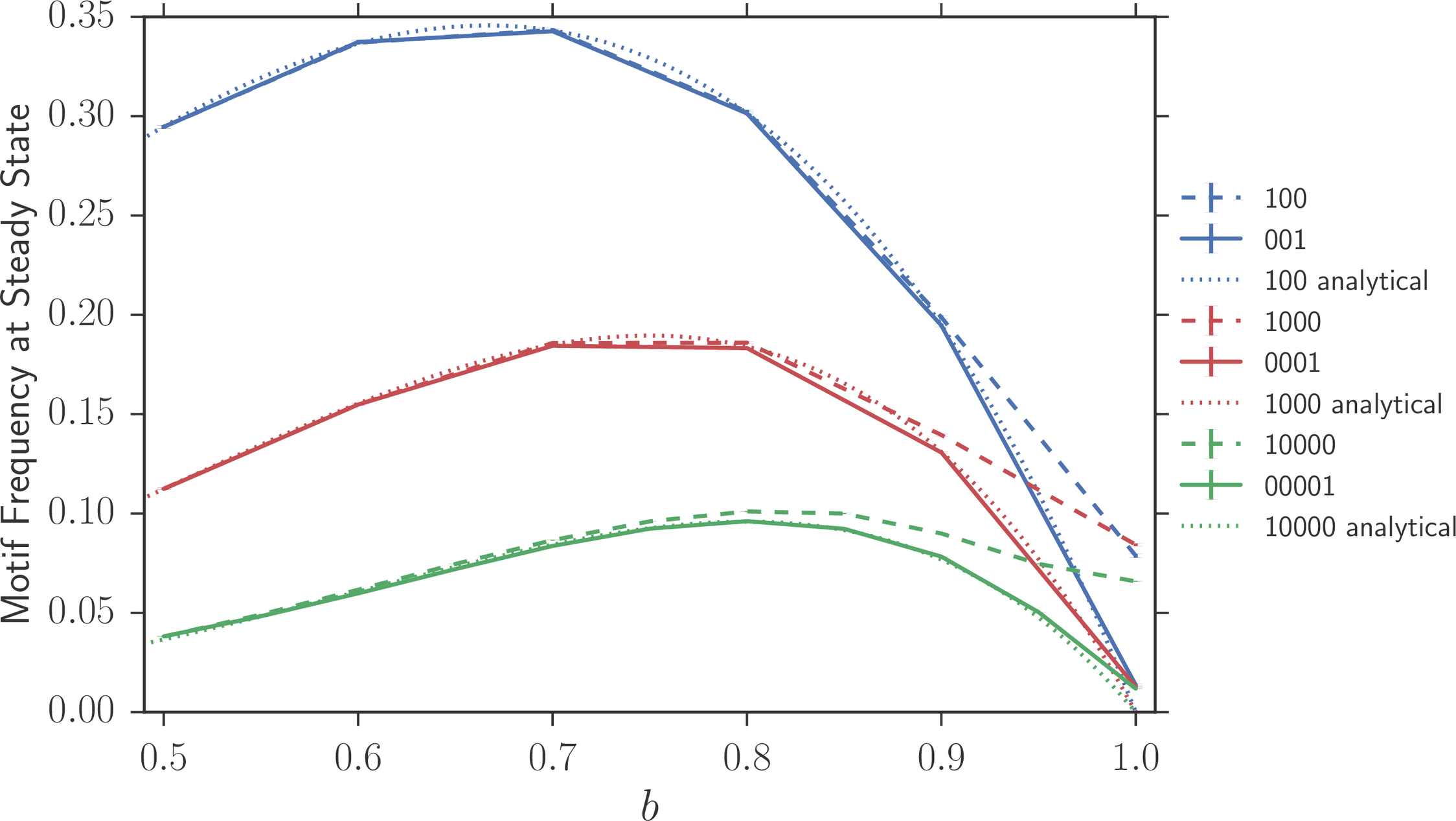

Supplement: S8 Fig — Smaller motifs are more abundant. The asymmetry in abundance between primed and non-primed motifs is preserved (for high biases). Dotted lines denote the approximation. These simulations are performed with M = 100, N = 100, r = 0.05, L = 7. (TIF) [file pone.0180208.s008.tif]

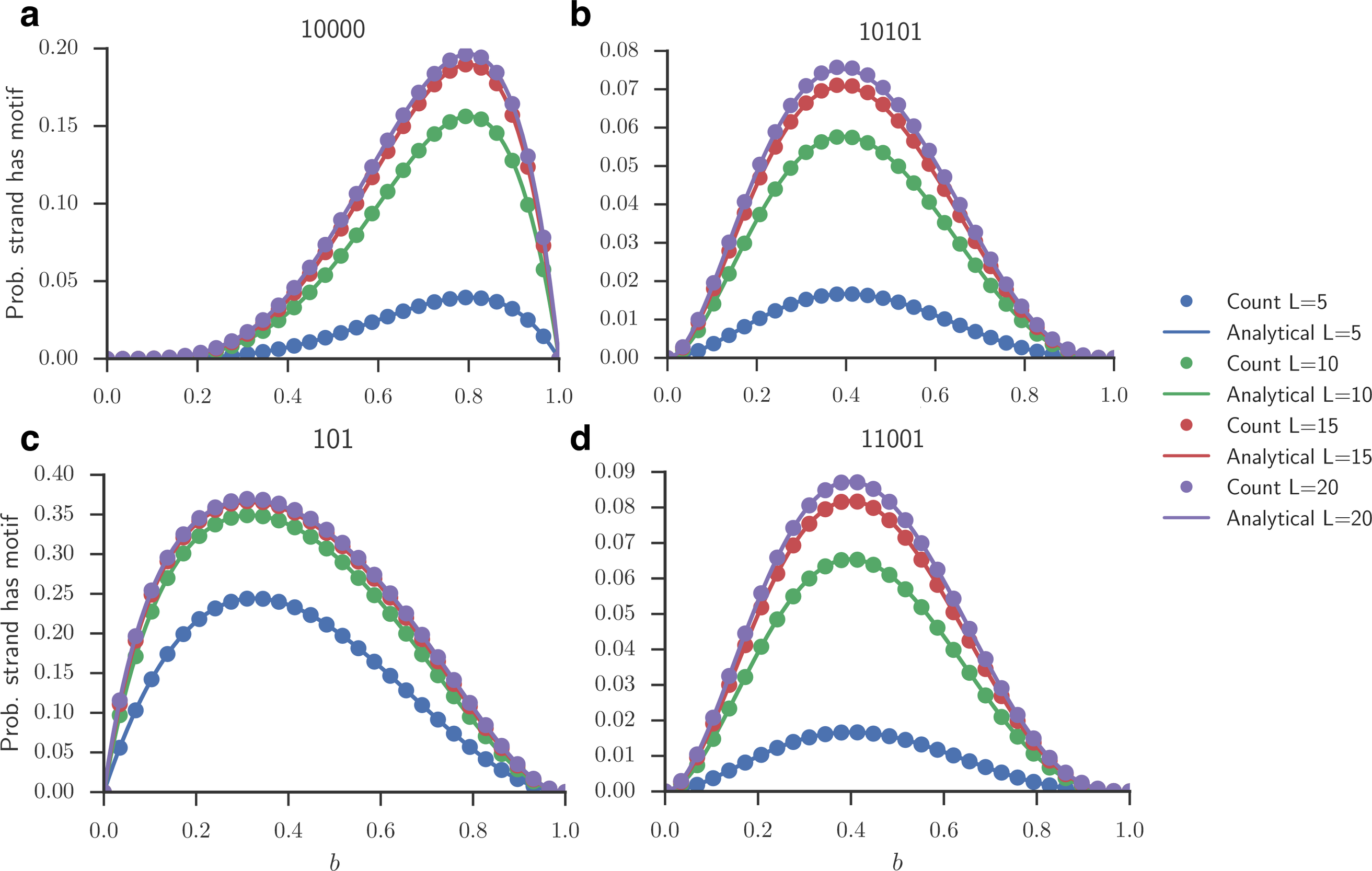

Supplement: S9 Fig — Lines represent the approximation for the probability that a strand contains a motif (Eq 3. in S1 File). To compute the probability of a motif per strand numerically, we generate all possible strands up to length L, count the number of motifs present and subsequently weight those by the probability that a strand reaches a particular length. Dots show the results for this computation. We show various maximum strand lengths and four different motifs with various patterns of overlaps: (a) 10000, (b) 10101, (c) 101, (d) 11001. Strand length distribution was calculated using r = 0.05, N = 100. (TIFF) [file pone.0180208.s009.tiff]
