# Supplementary material for: Prebiotic selection for motifs in a model of template-free elongation of polymers within compartments
Source: PLoS One. 2017 Jul 19;12(7):e0180208. doi: 10.1371/journal.pone.0180208 (PMC5516967; doi:10.1371/journal.pone.0180208)
Supplement: S1 File — We provide additional details for the simulations and the robustness of our results. We also provide an analytical approximation for the steady-state frequency of motifs. (PDF) [file pone.0180208.s010.pdf]

Supporting Information: Prebiotic selection for motifs in a model of template-free elongation of polymers within compartments

Simulation methods

Monte Carlo simulations were implemented in Python and run on Harvard's Odyssey computing cluster. Simulations were run until the system was at steady-state. We verified the steady-state condition empirically. To do so we start from a variety of initial conditions (both the limiting cases and intermediate (randomly chosen) initial conditions) and verify that they end up in the same state after 600 steps. We also check this by running the simulation for 40000 steps and verifying that the steady-state is indeed steady for large time scales. These results are summarized in S1 Fig. For computational efficiency, once we established these properties, we ran the simulations for 7000 steps.

For the single motif case, 50 replicate trials of 7000 rounds were run for the entire set of motifs of length 5, and biases b from 0 to 1 (step size 0.05). Steady-state motif frequencies for each trial were calculated by taking the mean of values pulled from after round 1000, and then the overall mean used in the figures is the average of the steady state frequencies for each trial. In the two-motif simulations, we ran 50 replicate trials of 15000 rounds each, using rounds after 1000 for steady-state calculations. Elongation probabilities of $r = 0.0125, 0.025, 0.05, 0.1, 0.2$ were used. In the analysis of elongation data during the creation of motif elongation pattern, only 15 trials were used for computational tractability. In the analysis of motif inter-arrival times, 25 replicates of 4000 rounds starting from the initial conditions of one population run at time 2000 were used.

To verify the simulation results in the main text, we also carry out the commonly used stochastic chemical kinetics simulation method, the Gillespie algorithm [1]. We pick rate constants such that there is on average 1 division for every rNM elongations, meaning that the sum of all the rates of division is 1 and the sum of the rates for the elongation reactions is rNM. The time that a particular reaction takes is exponentially distributed with mean $\frac{1}{1+rNM}$, which is the inverse of the sum of the rates of reaction. We observe close agreement with the results of both simulation methods. Gillespie simulations were run for approximately the same amount of time, until 7000 or 15000 compartment divisions occurred (we only recorded the state of the system following an elongation event, similar to the simulation of the main text), for the single and two-motif cases, respectively.

Analytical approximation of strand length distribution

We provide an approximation for the strand length distribution (with maximum length $L = \infty$). Let us consider the the probability that a particular strand is elongated λ times. This is a binomial distribution because in each time step, either the strand elongates (with probability r) or it does not. The number of rounds that a strand can possibly elongate is geometrically distributed with parameter $\frac{1}{N+1}$, which is the probability that the strand's host compartment is killed. Thus, we have the strand length distribution $\Lambda \sim \mathrm{Bin}\,(D, r)$ where $D \sim \mathrm{Geom}\left(\frac{1}{N+1}\right)$, where Λ is a random variable representing the length of a strand and D is a random variable representing the number of steps until the strand's (host cell's) death. Let us derive the probability distribution of Λ. To show

this in general (using simpler notation), let $\Lambda \sim \text{Bin}\,(D, r)$ and $D \sim \text{Geom}\,(g)$:

$$P\,(\Lambda = \lambda) = \sum_{d=\lambda}^{\infty} P(\Lambda = \lambda | D = d) P(D = d) = \sum_{d=\lambda}^{\infty} \binom{d}{\lambda} r^{\lambda} (1-r)^{d-\lambda} (1-g)^{d} g$$
$$= g\left(\frac{r}{1-r}\right)^{\lambda} \sum_{d=\lambda}^{\infty} \binom{d}{\lambda} (1-r)^{d} (1-g)^{d} = g\left(\frac{r}{1-r}\right)^{\lambda} \sum_{d=\lambda}^{\infty} \binom{d}{\lambda} [(1-r)(1-g)]^{d}$$

By ordinary generating function lemma, $\sum_{d=\lambda}^{\infty} \binom{d}{\lambda} x^{d} = \frac{x^{\lambda}}{(1-x)^{\lambda+1}}$ for fixed λ, we have:

$$P\,(\Lambda = \lambda) = g\left(\frac{r}{1-r}\right)^{\lambda} \frac{[(1-r)(1-g)]^{\lambda}}{[1-(1-r)(1-g)]^{\lambda+1}} = \frac{g r^{\lambda} (1-g)^{\lambda}}{(1-(1-r)(1-g))^{\lambda+1}}$$
$$= \frac{g r^{\lambda} (1-g)^{\lambda}}{(r + g(1-r))^{\lambda+1}} = \left(\frac{r(1-g)}{r+g(1-r)}\right)^{\lambda} \left(\frac{g}{r+g(1-r)}\right)$$

which is a Geometric distribution with parameter $\frac{g}{r+g(1-r)}$, thus $\Lambda \sim \text{Geom}\left(\frac{g}{r+g(1-r)}\right)$, in general. Substituting in the parameters specific to our case $(g = \frac{1}{N+1})$:

$$\Lambda \sim \text{Geom}\left(\frac{\frac{1}{N+1}}{r + \frac{1}{N+1}(1-r)}\right)$$

Simplifying, we have:

$$\Lambda \sim \text{Geom}\left(\frac{1}{(N+1)r + (1-r)}\right) \tag{1}$$

which represents the strand length distribution if there is no maximum strand length ($L = \infty$). When a maximum strand length is imposed, the distribution is truncated, and all lengths above L contribute to the probability of observing a strand of length L, giving us:

$$P\,(\Lambda = \lambda | L, N, r) = \begin{cases} \left(\frac{r(1-\frac{1}{N+1})}{r+\frac{1}{N+1}(1-r)}\right)^{\lambda} \left(\frac{\frac{1}{N+1}}{r+\frac{1}{N+1}(1-r)}\right) & 0 \leq \lambda < L \\ 1 - \sum_{j=0}^{L-1} \left(\frac{r(1-\frac{1}{N+1})}{r+\frac{1}{N+1}(1-r)}\right)^{j} \left(\frac{\frac{1}{N+1}}{r+\frac{1}{N+1}(1-r)}\right) & \lambda = L \end{cases} \tag{2}$$

Ordinary generating function lemma

We want to show that $\sum_{n=\lambda}^{\infty} \binom{n}{\lambda} x^{n} = \frac{x^{\lambda}}{(1-x)^{\lambda+1}}$ for fixed λ. We know that:

$$\frac{1}{(1-x)^{\lambda+1}} = \sum_{n=0}^{\infty} \binom{n + (\lambda+1) - 1}{n} x^{n} = \sum_{n=0}^{\infty} \binom{n+\lambda}{n} x^{n}$$

We can change to summing over $n - \lambda$:

$$= \sum_{n-\lambda=0}^{\infty} \binom{(n-\lambda)+\lambda}{n-\lambda} x^{n-\lambda} = \sum_{n=\lambda}^{\infty} \binom{n}{n-\lambda} x^{n-\lambda}$$

Multiplying by x^{λ}, we have:

$$\frac{x^{\lambda}}{(1-x)^{\lambda+1}} = \sum_{n=\lambda}^{\infty} \binom{n}{n-\lambda} x^{n} = \sum_{n=\lambda}^{\infty} \binom{n}{\lambda} x^{n}$$

as desired.

Analytical approximation of steady-state motif frequency

In what follows we make the simplifying assumption that a biasing motif exists in the compartment for its entire lifetime. In other words, the strands that exist in the compartment before the motif arrives are ignored. This makes the calculations easier, and as we will see in the figures, provides further evidence that some motifs rely more heavily on primers than others.

Using the strand length distribution derived above, we can calculate the probability of observing a strand of length $\Lambda = \lambda$. Conditioning on this strand length, we can calculate the probability that a given strand contains the motif. This depends on the motif itself. Let ℓ denote the length of the motif and m_0 denote the number of 0 monomers in the motif.

$$P\left(\text{strand contains motif}|\ell, m_0, b, L, N, r\right) = \\ \sum_{\lambda=\ell}^{L} P\left(\Lambda = \lambda|L, N, r\right) \left[\left(\lambda - \ell + 1\right) b^{m_0} (1-b)^{\ell - m_0} \left(\sum_{k=0}^{\lambda-\ell} \binom{\lambda - \ell}{k} b^k (1-b)^{\lambda-\ell-k} \right) \right. \\ \left. + \sum_{\beta=2}^{\lfloor \frac{\lambda}{\ell} \rfloor} (-1)^{\beta+1} \binom{\lambda - \beta\ell + \beta}{\beta} b^{\beta m_0} (1-b)^{\beta(\ell - m_0)} \sum_{k=0}^{\lambda-\beta\ell} \binom{\lambda - \beta\ell}{k} b^k (1-b)^{\lambda - \beta\ell - k} \right]$$

The first term in the bracket computes the probability of a motif starting at a particular position within the strand, and the sum is explicit accounting of all other appendage monomers that are attached to a motif (this naturally sums to one). When $\lambda \geq 2\ell$ this method will count non-overlapping motifs, but we are only interested in -at least once- appearance of the motif. Thus, we use inclusion-exclusion to control for over-counting by factor β (i.e. $\beta = 2$ refers to double counting) in the second term. Note that the first term in the bracket is a specific case of the second term with $\beta = 1$. Simplifying:

$$P\left(\text{strand contains motif}|\ell, m_0, b, L, N, r\right) = \\ \sum_{\lambda=\ell}^{L} P\left(\Lambda = \lambda|L, N, r\right) \left[\sum_{\beta=1}^{\lfloor \frac{\lambda}{\ell} \rfloor} (-1)^{\beta+1} \binom{\lambda - \beta\ell + \beta}{\beta} b^{\beta m_0} (1-b)^{\beta(\ell - m_0)} \right]$$

But this is not a complete picture. In the calculation above, we ignore auto-correlation between motifs (we assume motifs don't overlap). This is obviously not true for all motifs. For example, the 00000 overlaps in 4 positions with itself. Writing a precise probability distribution of motif counts that accounts for overlaps requires developing employing more mathematical machinery beyond the scope of our study. An interested reader can find deeper treatments in the literature [2, 3]. Instead, we develop a more concise approximation below, that is sufficient for our purposes.

To correct for overlaps, let Θ be the set of all possible lengths by which a motif overlaps by itself. Each entry of this set is a pair of values ϑ, ϑ_0 that represents the length of the overlap and the number of 0 monomers in that overlap, respectively. For instance, 10101 can overlap itself by length 0,1, or 3 (with 0,0, and 1 representing the number of zero monomers in these overlaps) - giving us the set $\Theta = \{(0,0), (1,0), (3,1)\}$.

Let α represents the number of distinct motif-containing runs in a strand and β represents the number of motifs present. For example, given motif 010 and strand 01010100010, we have $\alpha = 2$ and $\beta = 4$. Using this, we can capture the probability that

a strand contains the motif via:

$$P\left(\text{strand contains motif}|\ell, m_0, \Theta, b, L, N, r\right) = \\ \sum_{\lambda=\ell}^{L} P\left(\Lambda = \lambda | L, N, r\right) P\left(\text{strand contains motif}|\lambda, \ell, m_0, \Theta, b\right) \tag{3}$$

Explicitly expanding for the term $P\left(\text{strand contains motif}|\lambda\right)$, we have:

$$P\left(\text{strand contains motif}|\lambda, \ell, m_0, \Theta, b\right) = \\ \sum_{\vartheta, \vartheta_0 \in \Theta} \begin{cases} \sum_{\beta=1}^{\left\lfloor \frac{\lambda}{\ell} \right\rfloor} (-1)^{\beta+1} \binom{\lambda-\beta\ell+\beta}{\beta} b^{\beta m_0} (1-b)^{\beta(\ell-m_0)}, & \vartheta = 0 \\ \sum_{\alpha=1}^{1+\left\lfloor \frac{\lambda-(\ell-\vartheta)}{\ell} \right\rfloor} \sum_{\beta=\alpha+1}^{\alpha+\left\lfloor \frac{\lambda-\alpha\ell}{\ell-\vartheta} \right\rfloor} (-1)^{\beta+1} \binom{\lambda-\alpha\ell-(\beta-\alpha)(\ell-\vartheta)+\alpha}{\alpha} \alpha^{\beta-\alpha} & \\ \quad \times b^{\alpha m_0 + (\beta-\alpha)(m_0-\vartheta_0)} (1-b)^{\alpha(\ell-m_0)+(\beta-\alpha)(\ell-m_0-(\vartheta-\vartheta_0))}, & \vartheta > 0 \end{cases} \tag{4}$$

We use this formula to compute the probability that a strand made under bias b contains a motif (see S9 Fig. for a numerical verification). To find the overall motif frequency in the population, we first calculate the probability that a cell contains the motif assuming no motifs are initially present (under $b = 0.5$). This probability is the same as the expected frequency of cells containing the motif:

$$P\left(\text{cell has motif}|\ell, m_0, \Theta, b = 0.5, L, N, r, M\right) = \\ \mathbb{E}\left[\text{freq. of cells with motif}|\ell, m_0, \Theta, b, L, N, r, M\right] = \\ 1 - \left(1 - P\left(\text{strand contains motif}|\ell, m_0, \Theta, b = 0.5, L, N, r\right)\right)^M \tag{5}$$

Assuming that the contents of this cell are fully biased by the motif (ignoring primer effects), we have the expected frequency of motif-containing strands:

$$\mathbb{E}\left[\text{freq. of strands with motif}|\ell, m_0, \Theta, b, L, N, r, M\right] = \\ \mathbb{E}\left[\text{freq. of cells with motif}|\ell, m_0, \Theta, b, L, N, r, M\right] \times P\left(\text{strand contains motif}|\ell, m_0, \Theta, b, L, N, r\right) \tag{6}$$

We use this formula as our base case for comparing the difference in motif frequencies when population history effects are absent (or present). Here, by population history we refer to the primers or motifs made under no bias. We expect this approximation to be good for sufficiently large N and M for a fixed r. For small M and N, population history is likely to matter for all motifs because a significant fraction of strands are generated under no motif influence. For sufficiently large N and M (such as $N = M = 100$, and $r = 0.05$), we expect this calculation to match the frequency of the motifs that do not rely on primers. This is indeed the case as it can be seen in the Fig. 3 and S6-S8 Figs.

References

1. Gillespie DT. Exact stochastic simulation of coupled chemical reactions. The journal of physical chemistry. 1977;81(25):2340–2361.

2. Nuel G, Regad L, Martin J, Camproux AC. Exact distribution of a pattern in a set of random sequences generated by a Markov source: applications to biological data. Algorithms for Molecular Biology. 2010;5(1):15.

3. Fu JC. Distribution theory of runs and patterns associated with a sequence of multi-state trials. Statistica Sinica. 1996;p. 957–974.
